# Development and validation of a risk prediction model for anxiety or depression among patients with chronic obstructive pulmonary disease between 2018 and 2020

**DOI:** 10.1080/07853890.2022.2105394

**Published:** 2022-08-02

**Authors:** Tingyu Tang, Zongju Li, Xiaoling Lu, Jianzong Du

**Affiliations:** a Department of Respiratory Medicine, Zhejiang Hospital, Hangzhou, China; b The Second Clinical Medical College, Zhejiang Chinese Medical University, Hangzhou, China

**Keywords:** Prediction model, validation, anxiety, depression, COPD

## Abstract

Anxiety and depression are important risk factors for chronic obstructive pulmonary disease (COPD). The aim of this study was to develop a prediction model to predict anxiety or depression in COPD patients. The retrospective study was conducted in COPD patients receiving stable treatment between 2018 and 2020 to develop prediction model. The variables, were readily available in clinical practice, were analysed. After data preprocessing, model training and performance evaluation were performed. Validity of the prediction model was verified in 3 comparative model training. Between 2018 and 2020, 375 eligible patients were analysed. Thirteen variables were included into the final model: gender, age, marital status, education level, long-term residence, per capita annual household income, payment method of medical expenses, direct economic costs of treating COPD in the past year, smoking, COPD progression, number of acute exacerbation of COPD in the last year, regular treatment with inhalants and family oxygen therapy. Risk score threshold in each sample in the training set was 1.414. The area under the curve value was respectively 0.763 and 0.702 in the training set and test set, which were higher than three comparative models. The simple prediction model to predict anxiety or depression in patients with COPD has been developed. Based on 13 available data in clinical indicators, the model may serve as an instrument for clinical decision-making for COPD patients who may have anxiety or depression.

**Key messages**
- Thirteen variables were included into the prediction model.
- The AUC value was, respectively, 0.763 and 0.702 in the training set and test set, which were higher than three comparative models.
- The simple prediction model to predict anxiety or depression in patients with COPD has been developed.

## Introduction

Chronic obstructive pulmonary disease (COPD) is predicted to become the third leading cause of death by 2030 [1]. It is characterized by airflow obstruction that leads to slowly progressive symptoms of persistent cough, wheezing and exertional dyspnoea. COPD also results in some extrapulmonary comorbidities, such as skeletal muscle dysfunction, cardiovascular disease, anaemia, diabetes and osteoporosis [2,3]. Anxiety is related to physical and psychological discomfort. Depression is accompanied by the high degree of emotional distress [4]. Anxiety and depression often co-occur. At least half of people with depression also have anxiety [5].

It is estimated that the prevalence of anxiety in COPD patients is 16%–31% [6,7]. Anxiety in COPD patients is related to increased morbidity and mortality, including more exacerbations, more functional limitations and longer hospital stays [6,8–13]. In addition, numbers of studies have reported that depressive symptoms in patients with COPD have adverse effects on functional mobility and mortality [14–17]. According to previous reports, more than one-third of COPD patients have symptoms of both anxiety and depression [5,18,19]. Some factors may contribute to the increase in the prevalence of depression in COPD patients, including low lung function, disease severity, severe dyspnoea, frequent hospitalisation, long-term oxygen therapy, gender, low body mass index, current smoking and social isolation [20–24].

Although certain interventions can improve health outcomes, the diagnosis of anxiety and depression in COPD patients is often unrecognized and untreated [22,25]. In addition, data collected in clinical practice are rarely used for prognosis prediction of anxiety and depression in COPD patients. Furthermore, studies on prediction models for anxiety and depression in patients with COPD are limited. In view of this, a risk prediction model for anxiety and depression in COPD patients was developed and validated. The clinical prediction tool may help decision-making and optimize psychological care for COPD patients.

## Methods

### Patients

The retrospective study was performed in Zhejiang hospital in Hangzhou, Zhejiang Province, China. A total of 375 patients diagnosed with COPD were enrolled between January 2018 and December 2020. Detailed inclusion criteria were as follows: (1) the diagnostic criteria were in line with the Guidelines for diagnosis and treatment of COPD formulated by the Global Initiative for Chronic Obstructive Pulmonary Disease; (2) patients receiving stable treatment; (3) patients aged from 40 to 80 years old; (4) patients were willing to participate in the study and sign informed consent. Detailed exclusion criteria were as follows: (1) patients with acute exacerbation of COPD or co-occurrence of other chronic lung diseases such as asthma, active pulmonary tuberculosis, lung cancer, bronchiectasis, pulmonary fibrosis, primary pulmonary arterial hypertension, interstitial lung disease or other active lung diseases; (2) patients with severe acute episodes of hemodynamic instability and co-occurring chronic disease; (3) patients had schizophrenia and other mental disorders based on detailed mental examination, routine scales and auxiliary examinations; (4) patients were treated with immunosuppressants, heparin, antiepileptics, aluminium and some drugs that may cause anxiety/depression symptoms; (5) patients with a diagnosis of anxiety/depression disorder prior to receiving treatment for COPD and COPD patients receiving anti-anxiety/depression therapy; (6) patients with substance dependence. According to the World Health Organisation International Classification of Diseases, 10th edition, Hamilton Depression Scale and Hamilton Anxiety Scale were used for psychological evaluation of COPD patients [26]. In addition, clinical interviews with mental health doctors were conducted to diagnose the combination of anxiety and/or depression in COPD patients. Among enrolling 375 COPD patients, 308 patients had depression or anxiety. Clinical diagnostic information and social statistical survey of 375 enrolled patients were collected by uniformly trained respiratory physicians to ensure the homogeneity of the information collection. This study was approved by the Medical Ethics Committee of Zhejiang Hospital (approval no. 2019-8 K).

### Data preprocessing

In this study, the number of samples was 375. Each sample corresponded to 27 clinical indicators, including body mass index, COPD progression, forced expiratory volume in 1 s (FEV1), FEV1/forced vital capacity (FVC), expected value of FEV1%, chronic obstructive pulmonary disease assessment test (CAT) score, drug information, family oxygen therapy, number of acute exacerbation of COPD in the last year, gender, age, marital status, education level, long-term residence, per capita annual household income, payment method of medical expenses, direct economic costs of treating COPD in the past year, smoking and other comorbidities (pulmonary arterial hypertension, coronary heart disease, heart failure, diabetes, arrhythmia, stroke, Parkinson’s disease, cancer and chronic kidney disease). By counting the number of missing samples of clinical indicators in each sample, the indicators with more than 100 missing samples were deleted, including weight, height, body mass index, smoking time, average number of cigarettes smoked per day, smoking cessation, quit smoking time, FEV1, expected value of FEV1%, FEV1/FVC and other comorbidities. The remaining clinical indicators with missing values were filled in. It is noted that clinical indicators of CAT score, anxiety scale and depression scale was not used in the model analysis. The reason was that the phenotype of depression or anxiety was inferred from these three clinical indicators. In the sample data, each sample corresponds to a label indicating whether the corresponding COPD patient has depression or anxiety. Based on the above conditions, 67 COPD patients without depression or anxiety and 308 COPD patients with depression or anxiety were counted. Each of the remaining samples corresponded to 13 indicators: gender, age, marital status, education level, long-term residence, per capita annual household income, payment method of medical expenses, direct economic costs of treating COPD in the past year, smoking, COPD progression, number of acute exacerbation of COPD in the last year, regular treatment with inhalants and family oxygen therapy. Samples (67) without anxiety and depression were used as normal controls. The other samples (308) were used as the samples with depression or anxiety. Clinical data were labelled as follows: For gender field, 1 and 2 represented female and male, respectively. For marital status field, 1–3 represented death of a spouse, single/divorced and married/de facto married, respectively. For education level field, 1–4 represented primary school or no primary school education, junior high school, high school or technical secondary school and college or above, respectively. For long-term residence field, 1 and 2 represented country and cities/towns, respectively. For per capita annual household income field, 1–4 represented 20,001–30,000, 30,001–40,000, 40,001–50,000 and ≥50,001, respectively. For payment method of medical expenses field, 1–4 represented public expense, health insurance, new rural cooperative medical system and self-paying, respectively. For direct economic costs of treating COPD in the past year field, 1–4 represented ≤3000, 3001–6000, 6001–9000 and ≥9001, respectively. For smoking field, 1 and 2 represented no and yes, respectively. For COPD progression field, 1–4 represented 0–5 years, 5–10 years, 10–15 years and >15 years, respectively. For number of acute exacerbation of COPD in the last year field, 1 and 2 represented <2 times and ≥2 times, respectively. For regular treatment with inhalants field, 1 and 2 represented no and other options, respectively. For home oxygen therapy field, 1 and 2 represented no and yes, respectively.

### Model training and performance evaluation

All samples were split according to the ratio of the training set:test set = 7:3. Random seed was set to eight. In the training set, logistic regression analysis was performed on the samples. The variables were optimized by stepwise forward selection method. The model was used to obtain the risk score of each sample. ROC analysis was performed on the risk score of each sample to obtain the risk score threshold. The performance evaluation was performed on the training set and test set.

### Sample size calculation and statistical analysis

**Figure 1.**
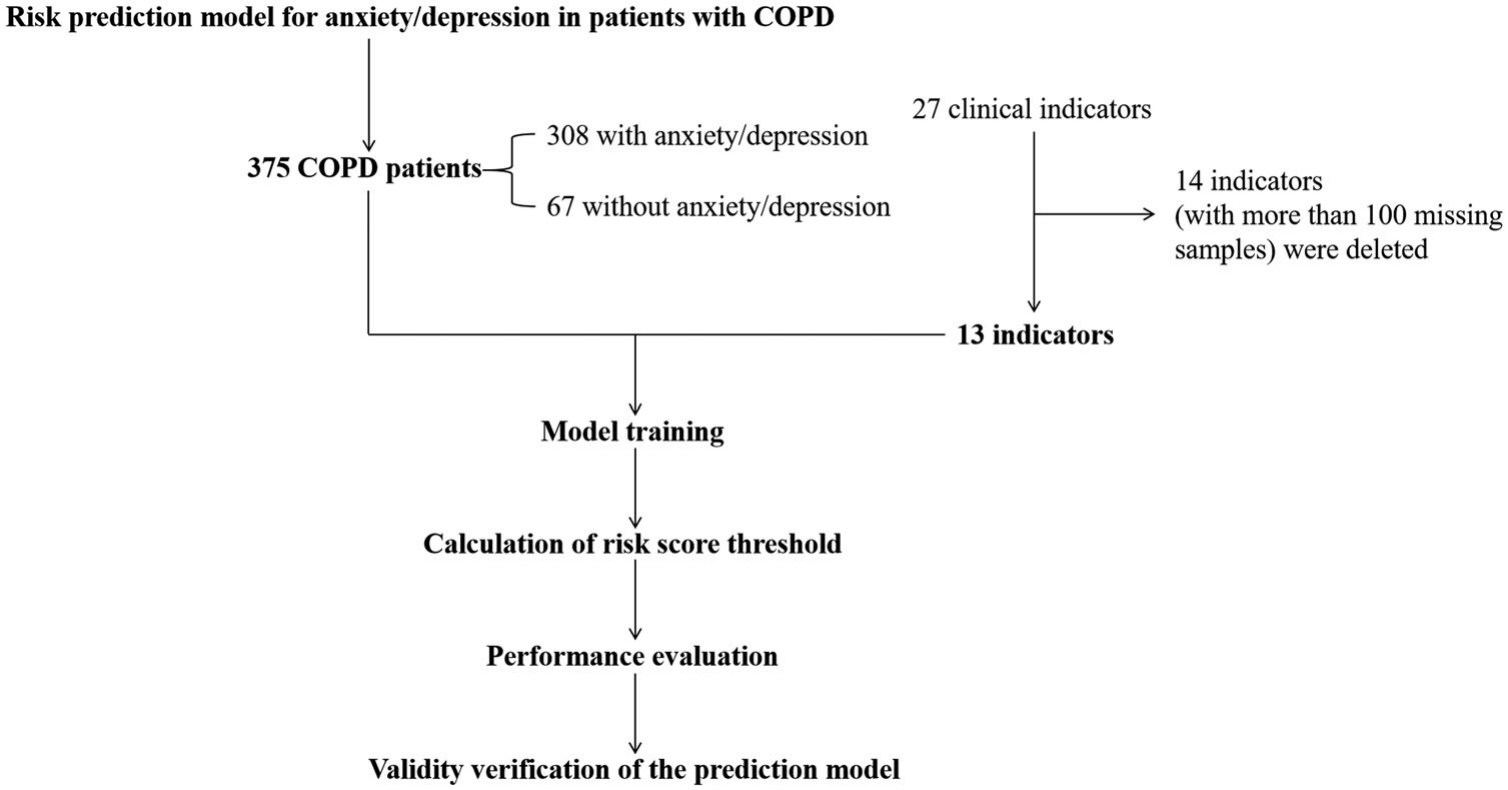
The flow chart of all methods in this study.

The formula *N* = *Z*^2^ * *P* (1 – *P*)/*E*^2^ was used for sample size calculation [27]. *Z* is the statistic. The confidence degree is set at 90%, Therefore, *Z* = 1.64. *E* is the error value. In this study, *E* = 5%. *P* is the prevalence rate (40%) of anxiety/depression in COPD patients. Combined with the patient shedding rate and other factors, 400 COPD patients were finally determined to be included in this study. The statistical test of clinical information for enrolled COPD patients was performed using compareGroups package in R language [28]. Categorical variables were analysed using Chi’s-square test. In the baseline feature table, space in the classification variables represents missing values. The results of classification variables are displayed in the form of frequency and percentage. The flow chart of all methods in this study is shown in Figure 1.

## Results

### Clinical information

A total of 375 COPD patients admitted between 2018 and 2020 were available for model development. Detailed information of these patients is listed in Table 1. The clinical indicators of age, payment method of medical expenses and smoking were significantly different in the anxiety and depression group.

**Table 1.** Clinical information of 375 COPD patients.

	Totality	COPD + anxiety/depression	COPD	
Clinical indicators	(*n* = 375)	(*n* = 308)	(*n* = 67)	*p* Value
Gender				.179
Female	88 (23.5%)	77 (25.0%)	11 (16.4%)	
Male	287 (76.5%)	231 (75.0%)	56 (83.6%)	
Age (years)				.031
	11 (2.9%)	11 (3.6%)	0 (0.0%)	
<70	57 (15.2%)	42 (13.6%)	15 (22.4%)	
≥90	31 (8.3%)	30 (9.7%)	1 (1.5%)	
70–80	116 (30.9%)	93 (30.2%)	23 (34.3%)	
80–90	160 (42.7%)	132 (42.9%)	28 (41.8%)	
Marital status				.452
Death of a spouse	24 (6.4%)	19 (6.2%)	5 (7.5%)	
Married/de facto married	348 (92.8%)	287 (93.2%)	61 (91.0%)	
Single/divorce	3 (0.8%)	2 (0.6%)	1 (1.5%)	
Education level				.454
High school/technical secondary school	53 (14.1%)	44 (14.3%)	9 (13.4%)	
Junior high school	78 (20.8%)	68 (22.1%)	10 (14.9%)	
Primary school/no primary school education	193 (51.5%)	153 (49.7%)	40 (59.7%)	
College or above	51 (13.6%)	43 (14.0%)	8 (11.9%)	
Long-term residence				.069
	1 (0.3%)	1 (0.3%)	0 (0.0%)	
Country	345 (92.0%)	279 (90.6%)	66 (98.5%)	
Cities/towns	29 (7.7%)	28 (9.1%)	1 (1.5%)	
Per capita annual household income (RMB)				.321
20,001–30,000	5 (1.3%)	5 (1.6%)	0 (0.0%)	
30,001–40,000	37 (9.9%)	33 (10.7%)	4 (6.0%)	
40,001–50,000	189 (50.4%)	149 (48.4%)	40 (59.7%)	
≥50,001	144 (38.4%)	121 (39.3%)	23 (34.3%)	
Direct economic costs of treating COPD in the past year (RMB)				.359
≤3000	226 (60.3%)	190 (61.7%)	36 (53.7%)	
3001–6000	114 (30.4%)	91 (29.5%)	23 (34.3%)	
6001–9000	16 (4.3%)	11 (3.6%)	5 (7.5%)	
≥9001	19 (5.1%)	16 (5.2%)	3 (4.5%)	
Smoking				.004
No	202 (53.9%)	177 (57.5%)	25 (37.3%)	
Yes	173 (46.1%)	131 (42.5%)	42 (62.7%)	
COPD progression (years)				.077
	1 (0.3%)	0 (0.0%)	1 (1.5%)	
>15	149 (39.7%)	126 (40.9%)	23 (34.3%)	
0–5	84 (22.4%)	66 (21.4%)	18 (26.9%)	
10–15	56 (14.9%)	42 (13.6%)	14 (20.9%)	
5–10	85 (22.7%)	74 (24.0%)	11 (16.4%)	
Number of acute exacerbation of COPD in the last year (times)				.817
	9 (2.4%)	7 (2.3%)	2 (3.0%)	
<2	253 (67.5%)	207 (67.2%)	46 (68.7%)	
≥2	113 (30.1%)	94 (30.5%)	19 (28.4%)	
Regular treatment with inhalants				.359
No	261 (69.6%)	218 (70.8%)	43 (64.2%)	
Yes	114 (30.4%)	90 (29.2%)	24 (35.8%)	
Home oxygen therapy				.985
No	344 (91.7%)	282 (91.6%)	62 (92.5%)	
Yes	31 (8.3%)	26 (8.4%)	5 (7.5%)	
Payment method of medical expenses				.011
Public expense	34 (9.1%)	33 (10.7%)	1 (1.5%)	
New rural cooperative medical system	10 (2.7%)	10 (3.2%)	0 (0.0%)	
Health insurance	315 (84.0%)	254 (82.5%)	61 (91.0%)	
Self-paying	16 (4.3%)	11 (3.6%)	5 (7.5%)	

RMB: renminbi.

### Model training

**Figure 2.**
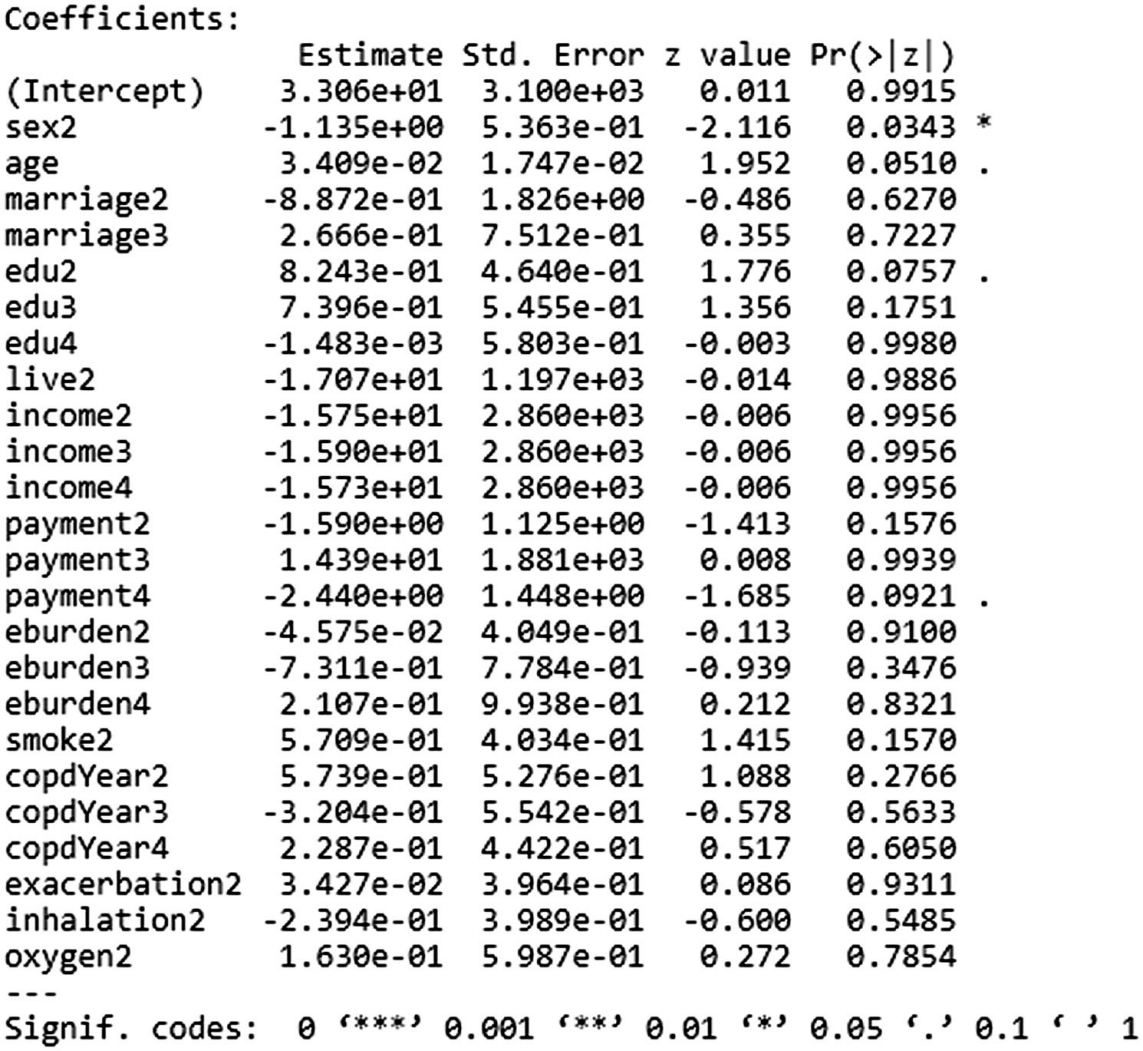
The result of model training.

Logistic regression analysis was performed on the samples in the training set. The variables were optimized by stepwise forward selection method. The final result of model training is shown in Figure 2. The above result was converted into the following formula:

*Y* = 33.06 + sex2*(–1.135)+age*0.03409 + marriage2*(–0.8872)+marriage3*0.2666 + edu2*0.8243 + edu3*0.7396 + edu4*(–0.001483)+live2*(–17.07)+income2*(–15.75)+income3*(–15.9)+income4*(–15.73)+payment2*(–1.59)+payment3*14.39 + payment4*(–2.44)+eburden2*(–0.04575)+eburden3*(–0.7311)+eburden4*0.2107 + smoke2*0.5709 + copdYear2*0.5739 + copdYear3*(–0.3204)+copdYear4*0.2287 + exacerbation2*0.03427 + inhalation2*(–0.2394)+oxygen2*0.163.

In the formula, *Y* and * represented the risk score and multiplier, respectively. The specific values of each indicator in the set of independent variables are as follows: sex2 = 0 and sex2 = 1 when gender is female and male, respectively. Age was the specific age value. Marriage2 = 0/1/0 and marriage3 = 0/0/1 when marital status is death of a spouse, single/divorced, married/de facto married, respectively. Edu2 = 0/1/0/0, edu3 = 0/0/1/3 and edu4 = 0/0/0/1 if the education level is primary school or no primary school education, junior high school, high school or technical secondary school and college or above, respectively. Live2 = 0 and live2 = 1 when long-term residence is country and cities/towns, respectively. Income2 = 0/1/0/0, income3 = 0/0/1/0 and income4 = 0/0/0/1 when the per capita annual income of households is 20,001–30,000, 30,001–40,000, 40,001–50,000 and ≥50,001, respectively. Payment2 = 0/1/0/0, Payment3 = 0/0/1/0, Payment4 = 0/0/0/1 when payment method of medical expenses is public expense, health insurance, new rural cooperative medical system and self-paying, respectively. Eburden2 = 0/1/0/0, eburden3 = 0/0/1/0 and eburden4 = 0/0/0/1 when direct economic costs of treating COPD in the past year are ≤3000, 3001–6000, 6001–9000 and ≥9001, respectively. When not smoking, smoke2 = 0. When smoking, smoke2 = 1. CopdYear2 = 0/1/0/0, copdYear3 = 0/0/1/0 and copdYear4 = 0/0/0/1 when COPD progression is 0 to 5 years, 5–10 years, 10–15 years and >15 years, respectively. Exacerbation2 = 0 and Exacerbation2 = 1 when number of acute exacerbation of COPD in the last year is <2 times and ≥2 times, respectively. Inhalation2 = 0 and inhalation2 = 1 when there is no regular inhalant treatment and other options, respectively. Oxygen2 = 0 when there is no home oxygen therapy. Oxygen2 = 1 when there is home oxygen therapy.

### Risk score threshold

In the training set, the risk score of each sample was obtained by using the model. Result of ROC analysis showed that risk score threshold for each sample was 1.414. If the risk score in each sample was ≤1.414, the COPD subject was judged not to have anxiety or depression. Or to say the risk of anxiety or depression was low. The risk of anxiety or depression was high if the risk score in each sample is >1.414.

### Performance evaluation

**Figure 3.**
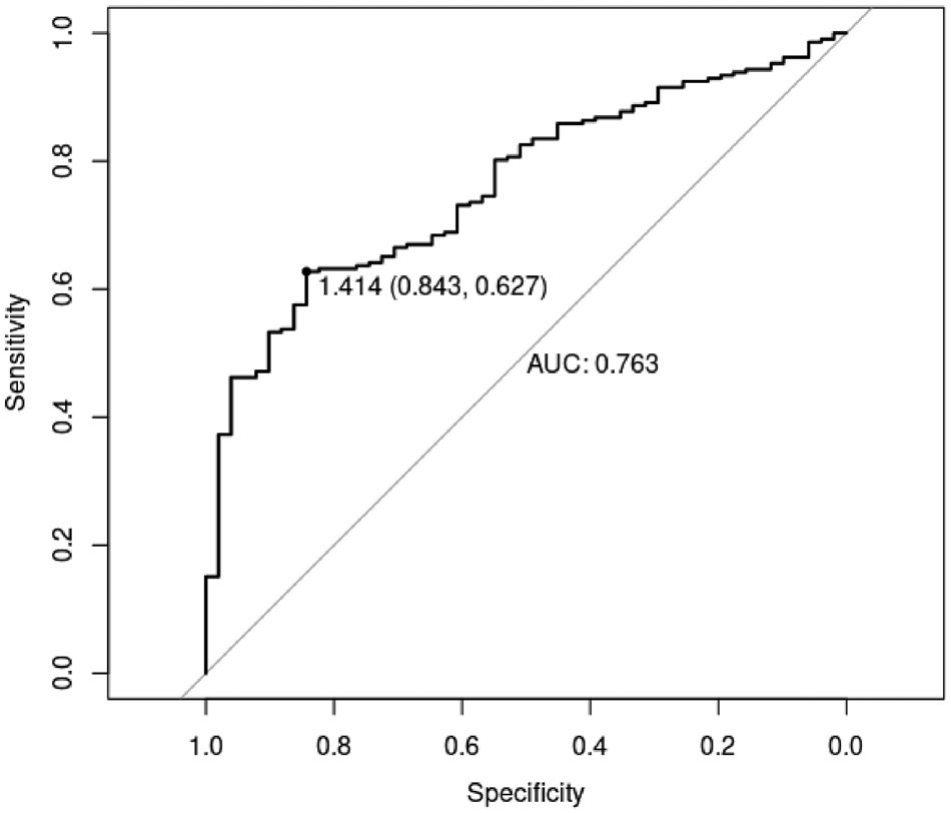
ROC curves of performance evaluation on the training set.

**Figure 4.**
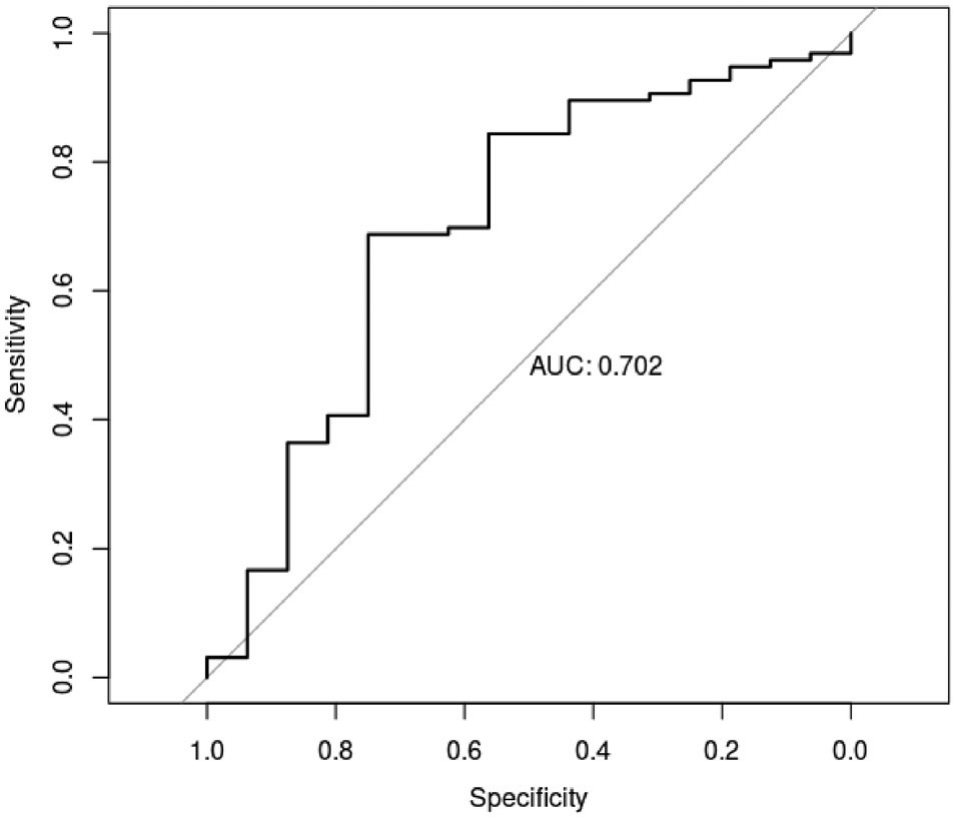
ROC curves of performance evaluation on the test set.

The result of performance evaluation on the training set is shown in Figure 3. It can be seen that the specificity and sensitivity of this model is 0.627 and 0.843, respectively. The area under the curve (AUC) value is 0.763. In addition, the performance evaluation was also performed on the test set (Figure 4). AUC value is 0.702. Due to the performance evaluation results of the model in the training set and test set based on 13 clinical indicators, the AUC values are all >0.7, indicating that the model has a good predictive effect on whether COPD patients suffer from depression or anxiety.

### Validity verification of the prediction model

**Figure 5.**
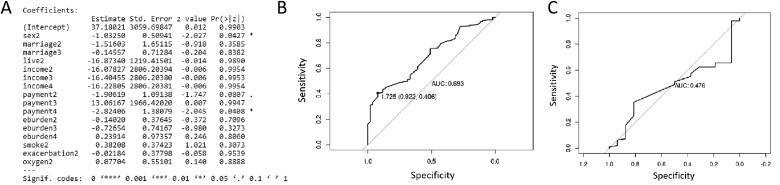
The result of model training (A), performance evaluation on the training set (B) and performance evaluation on the test set (C) in the first comparative model training.

**Figure 6.**
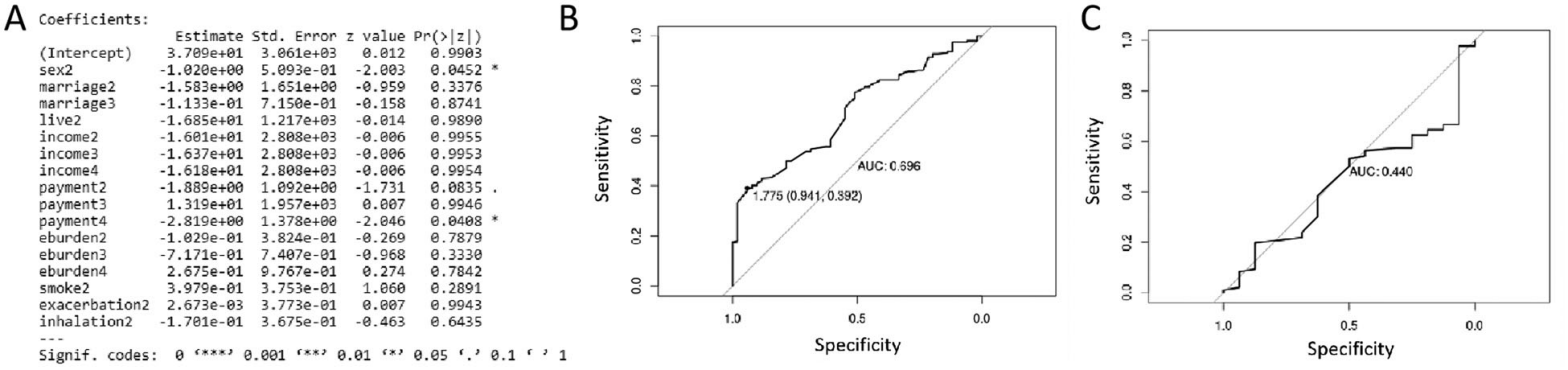
The result of model training (A), performance evaluation on the training set (B) and performance evaluation on the test set (C) in the second comparative model training.

**Figure 7.**
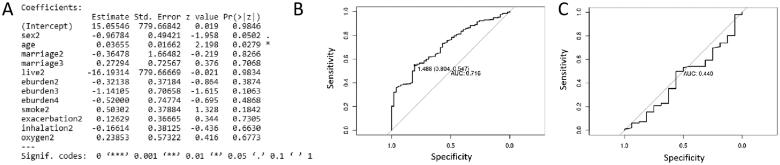
The result of model training (A), performance evaluation on the training set (B) and performance evaluation on the test set (C) in the third comparative model training.

To prove the prediction effect of the model, three comparative model training was redone on the basis of above model. In the first comparative model training, four variables including age, education level, COPD progression and regular treatment with inhalants were removed. The result of model training, performance evaluation on the training set and test set is shown in Figure 5(A–C), respectively. AUC value was 0.693 and 0.476 in the training set and test set, respectively. In the second comparative model training, four variables including age, education level, COPD progression and home oxygen therapy were removed. The result of model training, performance evaluation on the training set and test set is shown in Figure 6(A–C), respectively. AUC value was 0.696 and 0.440 in the training set and test set, respectively. In the third comparative model training, four variables including education level, COPD progression, per capita annual household income and payment method of medical expenses were removed. The result of model training, performance evaluation on the training set and test set is shown in Figure 7(A–C), respectively. AUC value was 0.716 and 0.440 in the training set and test set, respectively. In contrast, AUC value in three comparative models was lower (close to 0.5), which suggested that they have no clinical diagnostic value. In conclusion, the model based on 13 clinical indicators is a good predictor of depression or anxiety in COPD patients compared to comparative model.

## Discussion

In this study, a simple risk prediction model was developed to predict anxiety or depression in patients with COPD using routinely collected data in hospital, including gender, age, marital status, education level, long-term residence, per capita annual household income, payment method of medical expenses, direct economic costs of treating COPD in the past year, smoking, COPD progression, number of acute exacerbation of COPD in the last year, regular treatment with inhalants and family oxygen therapy. The prediction model can accurately predict anxiety or depression in COPD patients, with excellent diagnostic ability in internal validation and comparative model.

According to clinical information of these COPD patients, we found that male and the elderly (especially those over 80) were more likely to suffer from anxiety or depression. Gender differences exist when it comes to manifestation of depression, and can be found in prevalence rates, symptom profile and treatment response in COPD [29–31]. It is found that age is significant in explaining the life quality among patients with COPD [32]. The prevalence of COPD is variable between countries, overall there is a prevalence rate of about 10% in patients aged 40 and above [33]. In developing and developed countries, COPD is the most frequent respiratory disease in middle-aged and old people [34]. It is noted that the frequency of depression is determined according to age [35]. Moreover, older age has been considered as a predictor of caregiver depression in COPD patients [36].

It is reported that depression in COPD patients is related to marital status [14,37]. Education level is a risk factor of depressive and anxious symptoms in COPD patients [18]. It is shown that COPD patients with the bachelor degree have fewer depressive symptoms when compared to COPD patients with no education, elementary school, middle school and high school education [38,39]. Jemal et al. found that lack of access to critical resource like sanitary residence facilities is one of socioeconomic risk factors for patients with COPD [40]. When analysing the residence place, the majority of patients live in the cities [41]. In Spain, it exceeds 70% of COPD patients and the reasons most frequently associated with under-diagnosis are limited residence in rural areas [42]. Thus, it can be seen that marital status, education level and long-term residence can be important clinically predictive indicators of anxiety or depression for COPD patients.

It is reported that the COPD patients who had low family income tended to suffer from anxiety and depression [18]. Screening COPD patients for concomitant psychological distress are important as it is found to contribute to poorer health outcomes across a number of domains, including general greater economic burden [43]. Cost effectiveness is measured in one study of 224 patients with COPD [44]. At 12-month follow-up, expenses related to hospital admissions are reduced in the psychological therapy group. Maybe, per capita annual household income, payment method of medical expenses, direct economic costs of treating COPD in the past year can be used as potential predictive clinical indicators for anxiety or depression in patients with COPD.

It is supported that there is an association between COPD, depressive symptoms and smoking [45]. In addition, anxiety and depression interact with smoking produces stronger combined effects on mortality risk in patients with COPD [46]. It is suggested that clinicians should think more about screening for depressive symptoms among COPD patients who are actively smoking. In patients with COPD, depression is significantly related to disease progression [47]. It is reported that depression is an independent risk factor for mortality in COPD patients and is associated with the increased risk of exacerbations [11,48–50]. In addition, anxiety symptoms in COPD patients may distract patients from self-management of disease exacerbations [51]. COPD is related to occupational and environmental inhalants [42]. It is found that severe COPD patients had the higher risk of depression, with rates of depression up to 62% in oxygen dependent patients [52]. It is indicated that above clinical indicators can be taken into account to predict anxiety or depression in COPD patients.

## Conclusions

In conclusion, the model’s prediction capability is satisfactory in terms of screening anxiety or depression individuals from COPD patients. The prediction model may be used as a tool to help clinical doctors identify anxiety or depression patients and take a modulated approach to disease treatment. However, there are some limitations in our study. First, the prediction model is needed to be validated in large and independent populations. Second, the prediction model is needed to validate in an independent population from geographically different areas. Third, more pertinent medical information probably contributes to anxiety and depression, such as comorbid physical conditions, polypharmacy, younger age, living alone, unemployment, childhood trauma, female gender, psychiatric history and so on can be considered as variables, which can be included into the predictive model. Fourth, specific age groups and specific areas of study may be more meaningful for the predictive model, which may be considered in the further study.

In spite of the above limitations, the study may shed some light on clinical value in predicting patients’ psychological states. The risk prediction model is built from 13 readily available clinical indicators, which imply a straight-forward application in clinical practice.

## Abbreviations

AUC: Area under the curve
COPD: Chronic obstructive pulmonary disease
FEV1: Forced expiratory volume in 1s
FVC: Forced vital capacity
